# Exposure to Misinformation, Risk Perception, and Confidence towards the Government as Factors Influencing Negative Attitudes towards COVID-19 Vaccination in Malaysia

**DOI:** 10.3390/ijerph192214623

**Published:** 2022-11-08

**Authors:** Emma Mohamad, Jen Sern Tham, Siti Zaiton Mohd Ajis, Mohammad Rezal Hamzah, Suffian Hadi Ayub, Andi Muhammad Tri Sakti, Arina Anis Azlan

**Affiliations:** 1 Centre for Research in Media and Communication, Faculty of Social Sciences and Humanities, Universiti Kebangsaan Malaysia, Bangi 43600, Selangor, Malaysia; 2 UKM × UNICEF Communication for Development Centre in Health, Faculty of Social Sciences and Humanities, Universiti Kebangsaan Malaysia, Bangi 43600, Selangor, Malaysia; 3 Department of Communication, Faculty of Modern Languages and Communication, Universiti Putra Malaysia, Seri Kembangan 43400, Selangor, Malaysia; 4 Department of Communication, Faculty of Business and Communication, Universiti Malaysia Perlis, Kangar 01000, Perlis, Malaysia; 5 Faculty of Communication and Media Studies, Universiti Teknologi MARA, Shah Alam 40450, Selangor, Malaysia; 6 Faculty of Communication Science, Mercu Buana University, Jakarta 11650, Indonesia

**Keywords:** COVID-19, vaccination, government, cross sectional survey, perceived risk

## Abstract

Introduction: This study explored exposure to misinformation, COVID-19 risk perception, and confidence towards the government as predictors of negative attitudes toward the COVID-19 vaccine. Methods: A cross-sectional survey was carried out from 30 June to 30 August 2021 involving 775 respondents. The survey instrument for the questionnaire was an adaptation from various different studies consisting of five main variables: (1) misinformation about vaccination; (2) risk perception toward COVID-19; (3) attitudes toward the vaccination programme; (4) intention to get vaccinated; and (5) public confidence in the government in executing the vaccination programme. Results: The results of this study indicate that higher exposure to misinformation led to higher levels of negative attitudes toward the COVID-19 vaccine. When the perceived risk of COVID-19 infection was high, mistrust of vaccine benefits was low but there were also higher worries about the future effects of the vaccine. Confidence in the government was associated with lower negative attitudes toward the COVID-19 vaccine. Conclusion: The results of this study may help develop an understanding of negative attitudes toward vaccinations in Malaysia and its contributing factors.

## 1. Introduction

COVID-19 vaccinations have become crucial in supplementing individual preventive actions to combat the pandemic, and vaccination coverage is critical for maintaining efficient public health measures. Governments worldwide have made significant efforts to implement successful procurement and vaccination programmes for individuals since the availability of COVID-19 vaccines [1]. The severity of the COVID-19 pandemic on populations worldwide will be reduced significantly only if the worldwide vaccination deployment is successful. On the other hand, a vaccination programme is not without difficulties, particularly on a global scale. While the goal is to make the COVID-19 vaccine available and accessible to everyone, persuading people to vaccinate themselves is a different issue.

Nonetheless, due to the rapid process of vaccine development, various questions concerning vaccine acceptability and safety emerged as community concerns potentially influencing attitudes and behaviours toward vaccine hesitancy [2]. An earlier study proved how public negative attitudes toward the COVID-19 vaccine resulted in prominent resistance toward vaccination in the first phase of its introduction, even when the health authorities made it compulsory [3]. Among negative attitudes surrounding COVID-19 vaccine hesitancy were concerns about vaccine safety [4], worries about potential unforeseen side effects, a high level of mistrust of vaccine benefits [5], concerns of commercial profiteering [6], and preference towards natural immunity compared to the vaccine [7]. Therefore, to convince the public to agree to be vaccinated, trust must be built; information about the development of these vaccines must be made public so that people are aware and informed.

The significant growth of health information sources online has made it challenging for health authorities to ensure that accurate information reaches the public. Studies have documented the prevalence of misinformation on health-related issues such as vaccination, pandemic, non-communicable diseases, and medical treatment [8] and its role in diverting individuals from performing correct health behaviour, including preventive behaviour during the COVID-19 pandemic. Several factors, such as poor information infrastructure, lack of proper knowledge-sharing culture, and resistance to technology adaptation, remain the main challenges in dealing with misinformation [9]. Previous studies have shown that exposure to misinformation has led people to perform misguided COVID-19 preventive behaviours while discouraging them from performing the recommended ones [10]. Exposure to misinformation has also increased religious misinformation beliefs and conspiracy beliefs [11] and negatively impacted individuals’ mental health [12].

The prevalence of misinformation related to COVID-19 is high on social media and broadly delivered via online messaging services, making it an added challenge for the government to end the pandemic. Moreover, inaccurate beliefs can also be caused by the government’s inability to clarify and provide trusted information to counter the misinformation [13], which often leads to mistrust toward the government. Studies have suggested that clear messages and knowledge dissemination were positively associated with trust in the government when introducing COVID-19-preventive behaviours [14]. In regard to vaccination intake, several studies also revealed how the element of mistrust—mistrust toward health authorities and healthcare workers [15], mistrust towards biomedical science [16], and mistrust in medical information while believing conspiracy theories [17]—is significantly associated with vaccine hesitancy.

Another factor associated with the decision to take the vaccine is risk perception. A previous study has shown that public intention to be vaccinated is influenced by their perceived likelihood of being infected and the potential adverse effect of contracting COVID-19 [18,19]. In turn, risk perception is influenced by factors such as incorrect beliefs spread on social media (e.g., COVID-19 is no more dangerous than influenza, and there is no need to wear a mask) [20], experience of COVID-19, mass media exposure, knowledge about COVID-19, and perceived mortality. Populations of low-to-middle-income countries experienced higher mortality rates due to COVID-19 [21] yet showed more willingness to take the COVID-19 vaccines as compared to populations of high-income countries [22].

In Malaysia, there has been a discrepancy between public confidence in national and state governments in handling vaccination programmes. It was reported that most Malaysians trust the federal government’s ability to curb COVID-19 through its vaccination programme [23], which resulted in a high vaccination rate. However, a study in Sabah revealed confidence and convenience as factors associated with vaccine hesitancy among Sabah populations, particularly among the self-employed and unemployed [24]. The study also showed religious belief (being a Muslim) as one of the demographic factors associated with vaccine hesitancy. Corroborating the above findings, Ruhi et al., through their study comparing vaccine hesitancy among West and East Malaysian populations, noted that religious restrictions make vaccine hesitancy more problematic in East Malaysia as compared to in West Malaysia [25]. The lack of public confidence in the government and community disagreement over the religious permissibility of vaccines in certain parts of this country has proven the lack of proper communication messaging and a system to counter the negative public perception towards vaccination. Even so, the opportunity to correct public misperception remains open as a study reported that many populations exposed to vaccine misinformation still want to acquire additional vaccine-related information to overcome their vaccine hesitation [26].

While many studies have examined the role of negative attitudes toward vaccine hesitancy [3,4,27], the present study aims to explore factors that influence an individual’s negative attitudes toward vaccination. It is hypothesised that exposure to misinformation, COVID-19 risk perception, and confidence towards the government are predictors to negative attitudes toward the COVID-19 vaccine. This study employed a cross-sectional survey that was carried out from 30 June to 30 August 2021 during the second phase of the COVID-19 lockdowns in Malaysia and also when the COVID-19 vaccinations were initially being made available to the public. The results of this study may help in developing an understanding of negative attitudes toward vaccinations in Malaysia and their contributing factors.

## 2. Materials and Methods

### 2.1. Study Design and Setting

This cross-sectional investigation was conducted from 30 June to 30 August 2021 during the first phase of the National Recovery Plan period in Malaysia. This study was funded by Universiti Kebangsaan Malaysia (UKM) through a matching grant collaboration with UNICEF in order to investigate exposure to misinformation, risk perception, and public confidence in the government on the COVID-19 vaccination programme. The study received ethical approval from the UKM Ethics Committee which covered the aspects of protocol, procedures, the information sheet, and the consent statement (JEP-2020-276). A total of 775 respondents were involved in the study, representing the Malaysian population with a ±5% margin of error and a confidence level of 95% [28,29].

### 2.2. Data Collection

The data were collected online using the Survey Monkey platform, and the invitation to participate in this study was voluntary. To participate, respondents were required to read the information sheet and give consent by clicking the ‘Continue’ button prior to answering the self-administered questionnaire. Members of the Malaysian public who participated in the study were above the age of 18 and were currently residing in the country. Several strategies were employed to reach the targeted number of respondents despite the MCO. Overall, the dissemination of the survey utilised various social media platforms (WhatsApp, Facebook, Twitter, and Instagram). Facebook and WhatsApp were most effective as the most popular social media platforms in Malaysia [30]. The researchers also reached out to numerous networks through emails and personal outreach. The message to the survey link and a general description of the survey and questionnaire was prepared in English and the Malay language, considering the multi-ethnic demographics in Malaysia.

### 2.3. Survey Questionnaire

The survey instrument for the questionnaire was an adaptation from various different studies. The questionnaire consisted of five main variables: (1) misinformation about vaccination; (2) risk perception toward COVID-19; (3) attitudes toward the vaccination programme; (4) intention to get vaccinated; and (5) public confidence in the government in executing the vaccination programme. Since the questionnaire was bilingual (English and Malay language), the study used a backwards-translation approach to translate the items between both languages. This was done to ensure linguistic and conceptual equivalence [31]. For validation of language constructs, bilingual arbiters were sought to consult and rectify any discrepancies on both versions.

To measure exposure to misinformation on vaccination, 10 items were adapted from previous research [32] using a Likert scale (1—‘Not at all’ to 4—‘Very frequently’). In order to measure risk perception toward vaccination, the respondents were asked to answer four questions adapted from previous studies [33,34,35]. The answer scale utilised was from 1—‘Not at all’ to 6—‘Completely’. Negative attitudes toward the vaccination programme were measured through four sub-domains: (i) mistrust of vaccine benefits (3 items); (ii) worries about unforeseen future effects (3 items); (iii) concerns about commercial profiteering (3 items); and (iv) preference for natural immunity (3 items). The Likert scale for these items ranged from 1—’ Strongly disagree’ to 6—‘Strongly agree’. Items for attitudes toward vaccination were adapted from past research [5,36]. To measure the intention of the Malaysian public to get vaccinated, 1 item was adapted from previous research [37] with a dichotomous answer scale (Yes or No). Finally, the measurement of public confidence was adapted from previous research [5] with 2 items. The Likert scale employed for both items was 1—‘No confidence’ to 6—‘Very high confidence’. Scores for the items in each variable were averaged to obtain total scores.

### 2.4. Statistical Analysis 

For this study, the data collected were analysed using the Statistical Package for the Social Sciences (SPSS), version 26. Descriptive analysis focused on frequencies and percentages for demographics; for inferential tests, the statistical significance level was set at *p* < 0.05. Internal consistency of the knowledge measures was tested using a reliability test, where the Cronbach’s alpha coefficient aided in determining the reliability of the variables. The results showed that the Cronbach’s alpha for misinformation (10 items) was 0.842. For risk perception (4 items), the Cronbach’s alpha was 0.676. For the four domains of attitudes toward the vaccination programme, (i) for mistrust of vaccine benefit (3 items), the Cronbach’s alpha was 0.878; (ii) for worries about unforeseen future effects (3 items), the Cronbach’s alpha was 0.769; (iii) for concerns about commercial profiteering (3 items), the Cronbach’s alpha was 0.812; and (iv) for preference for natural immunity (3 items), the Cronbach’s alpha was 0.786. The Cronbach’s alpha for public confidence was 0.833. This adds credence to the results as stated by Griethuijsen, Cronbach’s alpha values above 0.6 were considered adequate and reliable [38]. A hierarchical regression procedure was conducted to determine the relationships between selected demographics, exposure to misinformation, risk perception toward COVID-19, public confidence, and attitudes toward the vaccination programme.

## 3. Results

### 3.1. Descriptive Statistics

The main characteristics of the study population are reported in Table 1. The pool of respondents was 69.2% female and 30.8% male, with an average age of 33.71 years (SD = 10.71). Most of the respondents were ethnic Malay (67.5%), from Selangor and Kuala Lumpur (47.5%), lived in urban areas (64.9%), and worked in the private sectors (47%). Moreover, 54% of the respondents had income less than MYR 4360 per month or no income at all. The majority of the respondents reported good health status (84.6%), and 81.3% reported having no diseases at the time of the survey.

Table 2 presents the respondents’ primary sources of COVID-19 information. The most common information sources were government organisations, newspapers or online newspapers, and doctors or healthcare providers. Conversely, alternative medicine practitioners were the source least referred to by respondents when seeking COVID-19 information.

As shown in Table 3, respondents utilised online, social, and mainstream media to access information about COVID-19 vaccines. The majority of respondents preferred to use online news portals, Facebook, and television. Few respondents used radio and YouTube for news related to COVID-19 vaccines.

### 3.2. Exposure to Misinformation on COVID-19 Vaccination

Overall, the surveyed respondents were exposed to at least one kind of misinformation about COVID-19 vaccines (mean 1.81). Almost 60% of respondents reported that they were not exposed to misinformation related to COVID-19 vaccines affecting human DNA, COVID-19 vaccines containing pig fat (60.9%), and that COVID-19 vaccines can cause infertility in women (64%). The survey indicated that respondents were exposed (rarely, occasionally, and very frequently) to information about the COVID-19 vaccine causing severe side effects such as allergic reactions (82.5%); that COVID-19 vaccines cause serious side effects such as allergic reactions (62.1%); that a nurse fainted after she received the COVID-19 vaccine (58%); that COVID-19 vaccines contain live viruses that can make people sick with COVID-19 (46.7%); that once a person receives the COVID-19 vaccine, they will not have to wear a mask or practice social-distancing (42.4%); that those who have recovered from COVID-19 do not need to get vaccinated (40.9%); that COVID-19 vaccines affect human DNA (40.2%); that vaccines for COVID-19 have a microchip that can track the location of the patient (40%); that COVID-19 vaccines contain pig fat (39.2%); and that the COVID-19 vaccine can cause infertility in women (36%). This is presented in Table 4.

### 3.3. Risk Perception about COVID-19

The study found that 88% of respondents believed that COVID-19 is a problem that is important to them, and 80% indicated that they were worried about being infected with COVID-19 in the future (Table 5). However, only one-third of respondents (38.7%) believed they were likely to be infected with COVID-19 and felt at risk of COVID-19 infection (39.6%).

### 3.4. Attitudes toward the Vaccination Programme

A total of 82.2% of respondents agreed that they felt safe after being vaccinated. The majority of respondents (72.5% and 82.7%) agreed that they could rely on COVID-19 vaccines to stop serious infections and felt protected after getting vaccinated, respectively. Even so, respondents worried about unforeseen future effects of COVID-19 vaccines; the majority (81.7%) agreed that there might be problems with the vaccines that were currently unknown, although most of the vaccines appeared to be safe at the moment. Only 51.5% agreed that COVID-19 vaccines could cause unforeseen problems in children and 61.2% personally believed that there could be unknown long-term effects of the vaccine.

More than half of the respondents did not agree that vaccines make a large quantity of money for pharmaceutical companies but do not do much for regular people (63.7%); that authorities promote vaccination for financial gain, not for people’s health (81.4%); and that vaccination programmes are a big deception (89.6%). Moreover, the majority of respondents did not prefer natural immunity against COVID-19 infection, wherein 67.2% disagreed that natural immunity lasts longer than vaccination, 80% that natural exposure to viruses and germs gives the safest protection, and 82.9% that being exposed to diseases naturally is safer for the immune system than being exposed through vaccination (Table 6).

### 3.5. Public Confidence in Government and Willingness to Get Vaccinated

Slightly half of the respondents expressed their trust in the Malaysian government’s ability to manage the COVID-19 vaccination programme effectively (55.6%). However, more than half of the respondents believed that the Malaysian public health service effectively managed the COVID-19 vaccination program (72.3%). Regarding intention to get vaccinated, 99% of the respondents expressed their willingness to get vaccinated against COVID-19 (Table 7 and Table 8).

### 3.6. Ordinary Regression Analysis

Table 9 presents the results of regression models predicting four domains of negative attitudes towards the COVID-19 vaccine. Selected socio-demographic variables were controlled and entered in block one, while the main study variables were entered in block two. Overall, demographic variables accounted for a very small amount of variance in the four domains of negative attitudes towards COVID-19 vaccines (*R*^2^_Mistrust_ = 7.2%; *R*^2^_Worries_ = 4.3%; *R*^2^_Concerns_ = 13.2%; *R*^2^_Preference_ = 6.1%). More specifically, the results showed that age was positively associated with the four domains of negative attitudes towards COVID-19 vaccines (β_Mistrust_ = 0.23, *p* = 0.000; β_Worries_ = 0.17, *p* = 0.000; β_Concerns_ = 0.21, *p* = 0.000; β_Preference_ = 0.22, *p* = 0.000). Compared to females, males were positively associated with only two domains—concerns about commercial profiteering (β_Concerns_ = 0.16, *p* = 0.000) and preference for natural immunity (β_Preference_ = 0.09, *p* = 0.015). All ethnic groups were worried about unforeseen future effects of COVID-19 vaccines (β_Malay_ = 0.57, *p* = 0.003; β_Chinese_ = 0.51, *p* = 0.003; β_Indian_ = 0.20, *p* = 0.006; β_Bumiputera_ = 0.32, *p* = 0.006). Moreover, both Indians and Chinese had mistrust of vaccine benefits (β_Chinese_ = 0.53, *p* = 0.002; β_Indian_ = 0.18, *p* = 0.016) and had concerns about commercial profiteering of COVID-19 vaccines (β_Chinese_ = 0.57, *p* = 0.001; β_Indian_ = 0.51, *p* = 0.033). The results also revealed that income had a negative association with concerns about commercial profiteering of COVID-19 vaccines (β = −0.09, *p* = 0.022) and preference for natural immunity (β = −0.09, *p* = 0.042). 

After controlling the demographic variables, the main predictors accounted for 8%–21.3% of variation for the four domains of negative attitudes towards COVID-19 vaccines (*R*^2^_Mistrust_ = 15%; *R*^2^_Worries_ = 8.7%; *R*^2^_Concerns_ = 21.3%; *R*^2^_Preference_ = 8.3%). As predicted, exposure to COVID-19 misinformation was positively associated with four domains of negative attitudes toward COVID-19 vaccines (β_Mistrust_ = 0.11, *p* = 0.000; β_Worries_ = 0.13, *p* = 0.000; β_Concerns_ = 0.10, *p* = 0.003; β_Preference_ = 0.12, *p* = 0.001). Perceived risk had a negative relationship with mistrust of vaccine benefits (β = −0.07, *p* = 0.039) but had a positive relationship with worries about unforeseen future effects of COVID-19 vaccines (β = 0.10, *p* = 0.005). Moreover, people’s confidence in the government in managing the inoculation program was negatively associated with four domains of negative attitudes towards the COVID-19 vaccine (β_Mistrust_ = −0.26, *p* = 0.000; β_Worries_ = −0.12, *p* = 0.000; β_Concerns_ = −0.28, *p* = 0.000; β_Preference_ = −0.09, *p* = 0.017).

## 4. Discussion

The results of our study indicate that misinformation on COVID-19 is quite common, with respondents reporting that they have seen/read at least one inaccurate claim on the vaccine. Specifically, the claim that respondents were most exposed to was that the COVID-19 vaccine causes serious side effects such as allergic reactions. Corroborating the finding above, a previous study in the country suggested that public vaccination uptake is significantly influenced by the low risk of severe side effects [39]. Interestingly, misperception of the side effects of COVID-19 vaccination also happened to be the top predictor of vaccine hesitancy in other countries such as Egypt [40], the United States [41,42], and several countries in Europe [43]. Another false claim that the respondents were highly exposed to was that the vaccine is unsafe because it was developed rapidly. The rapid development of the COVID-19 vaccine has raised many concerns about its safety and efficacy [44]. The urgency to provide the vaccine within a short period of time has also resulted in a major challenge for the government to ensure transparency in the process of vaccine development [45]. Not only in Malaysia, but this false claim about vaccine safety is also common among unvaccinated populations in the United States, Canada, Sweden, and Italy [46].

In terms of risk perception, respondents felt that COVID-19 was an important issue for them, and worries that they would be infected in the future were very high. Additionally, the majority of respondents perceived that they would likely be infected with COVID-19 and have previously felt at risk of being infected. Several studies have also linked public COVID-19 risk perception with the willingness or hesitancy to get vaccinated. For instance, a study by Al-Qerem and Jarab suggested perceived risk of infection as a predictor of vaccination intention among the Middle Eastern population [47].

In the United States, it was proven that the vaccinated population showed a higher level of COVID-19 risk perception compared to those who were unvaccinated [48]. Additionally, in Malaysia, worry about being infected was also found to be a predictor of parental intention to vaccinate their children [49]. Therefore, increasing public perceived risk can be an imperative move to improve the population’s vaccine intake, in which the government may produce strategic regulations and the media can play its role to shape public perception.

The present study has also revealed that public confidence in the Malaysian government’s ability to manage the vaccination programme was high. This finding corroborates a past study conducted in Malaysia, which explained how the public had high trust in the government’s ability to manage the COVID-19 crisis in the beginning of the pandemic [50]. Studies conducted around the world have shown that although public confidence and trust in government are important to the success of vaccination programmes [51], many governments struggle with this. For instance, with a long history of vaccine hesitancy, the COVID-19 vaccination rate in Nigeria was reported as being very low due to public distrust toward the government [52]. In addition, a review study synthesising the determinants of COVID-19 vaccine hesitancy in South Africa reported public distrust as one of the predictors of low vaccination intake in the country [53]. Only 1% of respondents in the present study indicated that they would not take the COVID-19 vaccine. Comparatively, this rate is much lower than in other Southeast Asian countries such as Singapore (9.9%) [54], Thailand (10.2%) [55], and Indonesia (13.2%) [56].

When the COVID-19 vaccine became available to the public, there was a mix of reactions. Those who were hesitant were reported to believe that the vaccine is dangerous and useless, and COVID-19 is harmless, while those who were willing to be vaccinated were influenced by the number of COVID-19 cases and deaths in their respective locations [57]. The results of this study show that Malaysians held low levels of mistrust toward vaccine benefits, with many feeling safe and protected after taking the vaccine. Even so, there was a high level of worry about the unforeseen future effects of the vaccine. The same concern was common among the public in Pakistan [58] and the United States [59]. This sentiment is common in new medical developments such as treatment and vaccinations. One of them is a false claim that the mRNA genetic material in several vaccines can possibly alter human DNA [60]. In addition, aside from safety and efficacy, the rapid development of COVID-19 vaccines has also raised concerns about long-term effects, with no exception among healthcare workers [2]. Earlier studies documented a small percentage of healthcare workers who were hesitant to receive the COVID-19 vaccine [61,62,63].

Studies in the West have identified concerns of commercial profiteering and a preference for natural immunity as prominent factors leading to vaccine hesitation. In the UK, where 16% of the public indicated a high-level mistrust of the COVID-19 vaccine, many people expressed extreme negative attitudes relating to commercial profiteering and a preference for natural immunity [5]. This was not reflected in the Malaysian public. The present study found that most did not agree that pharmaceutical companies made a profit off the vaccines as compared to regular members of the public. The majority also did not agree that natural immunity was better than vaccines in protecting individuals against COVID-19 infection.

In general, the results of this study indicate that higher exposure to misinformation led to higher levels of negative attitudes toward the COVID-19 vaccine. When the perceived risk of COVID-19 infection was high, mistrust of vaccine benefits was low but there were also higher worries about the future effects of the vaccine. In other words, the Malaysian public trust that the vaccine will keep them protected from COVID-19 but are wary of its long-term effects. Previously, it was reported that a high level of COVID-19 vaccine acceptance in the country was due to the high perceived benefits of the vaccine, although many are still in doubt about the risks after being vaccinated [64]. In this study, confidence in government was associated with lower negative attitudes toward the vaccine across all four domains (mistrust of vaccine benefits, worries about unforeseen future effects, commercial profiteering, and preference for natural immunity). These findings support previous studies on the moderating effect of trust in the success of national vaccination programmes. A global survey reported respondents from China, South Korea, and Singapore who had a higher level of trust toward the government were more likely to get vaccinated [65].

In the global context, patterns of vaccine acceptance have been shown to be higher in countries with higher levels of perceived risk [47] and higher trust and confidence in the government [65]. A study conducted in South Asia showed similarities between antecedents to COVID-19 vaccine acceptance between four different countries in the region [66]. Additionally, a systematic review found that vaccination acceptance rates were highest in Ecuador, Malaysia, Indonesia, and China (above 90%); the lowest (below 60%) in Kuwait, Jordan, Italy, Russia, Poland, Italy, and France [67]. This alludes to the idea that countries with similar characteristics may share similar sentiments toward COVID-19 vaccinations and similar antecedents to vaccine acceptance. An exploration of these broader contexts is recommended.

## 5. Limitations

This study utilised a convenience sampling procedure via personal and professional networks of the researchers, disseminated through online/short messaging services. This strategy may have introduced bias as some groups may have been excluded with this method of sampling. As a result, the sample does not accurately reflect the overall population. However, as the data collection was performed during a national lockdown, it was deemed the best way possible to collect data given the limitations. When compared to the national demographics, the gender distribution of the sample does not accurately reflect the current Malaysian population. The respondents of the study consisted of 69.2% women, while the current Malaysian population estimates that only 49% of the population is female. In terms of racial distribution, the study had a similar percentage reflecting the two main races in the country; however, only 2.6% of respondents were Indian, while the current national statistics estimates 6.8% of the country’s population is Indian. In terms of the income distribution, 53.7% of respondents belonged to the below 40% income bracket, only 27.1% of respondents were in the middle 40% income bracket, and 19.2% of respondents came from the top 20% income bracket. This variation affects the representativeness of findings to the overall population.

Another limitation that any self-administered survey has is a social desirability bias among respondents. Respondents tend to answer questions on the basis of what they think will make them look good or what they perceive is the answer that other people expect from them. However, this study has tried to reduce this bias by assuring anonymised data collection and utilising online platforms.

## 6. Conclusions

This study explored factors that influence an individual’s negative attitudes toward vaccination. Findings showed that higher exposure to misinformation and perceived risk of COVID-19 infection led to higher negative attitudes toward the COVID-19 vaccine. This study also found that the public’s confidence in the government was high and associated with lower negative attitudes toward the vaccine across all four domains (mistrust on vaccine benefits, worries about unforeseen future effects, commercial profiteering, and preference for natural immunity).

## Figures and Tables

**Table 1:** Socio-demographic profiles (*n* = 775). ^a^ Mean ± standard deviation (range).

	Total	
	*n*	%
Socio-demographic:		
Gender		
Female	536	69.2
Male	239	30.8
Age	33.71 ± 10.71 (18–75) ^a^	
Ethnicity		
Malay	523	67.5
Chinese	167	21.5
Indian	20	2.6
Bumiputera (Sabah/Sarawak)	59	7.6
Others	6	0.8
Locality		
Urban	503	64.9
Rural	272	35.1
State		
Johor	60	7.7
Kedah	41	5.3
Kelantan	29	3.7
Melaka	19	2.5
Negeri Sembilan	44	5.7
Pahang	31	4.0
Perak	43	5.5
Perlis	6	0.8
Pulau Pinang	22	2.8
Terengganu	27	3.5
Sabah	30	3.9
Sarawak	41	5.3
Selangor	281	36.3
Federal Territory of Kuala Lumpur	87	11.2
Federal Territory of Putrajaya	1	0.1
Federal Territory of Labuan	13	1.7
Employment status		
Government employee	147	19.0
Private employee	364	47.0
Self-employed (registered)	35	4.5
Self-employed (not registered)	29	3.7
Unpaid family worker	4	0.5
Not employed	196	25.3
Income		
Under MYR 4360 per month (including no income)	416	53.7
MYR 4361–9620 per month	210	27.1
Above MYR 9621 per month	149	19.2
Health status		
Very bad	10	1.3
Bad	14	1.8
Average	96	12.4
Good	381	49.2
Very good	274	35.4
Health problem		
Yes, more than one disease	39	5.0
Yes, only one disease	106	13.7
No diseases	630	81.3

**Table 2:** Primary sources of COVID-19 information.

Source of Information	Frequency	Percentage
Government organisations	255	32.9
Newspaper or online newspapers	231	29.8
Doctor or healthcare provider	108	13.9
Brochures, pamphlets, etc.	62	8.0
Other	48	6.2
Friends/co-workers	34	4.4
Family	33	4.3
Alternative medicine practitioner	4	0.5

**Table 3:** Media platforms used for COVID-19 vaccine news.

Media	Percentage	
	Yes	No
Online news portal	69.0	31.0
Facebook	68.0	32.0
Television	64.4	35.6
WhatsApp	51.5	48.5
Instagram	39.5	60.5
Telegram	32.5	67.5
Twitter	31.6	68.4
Radio	29.4	70.6
YouTube	25.8	74.2

**Table 4:** Exposure to misinformation on COVID-19 vaccination.

	Total	
	*n*	%
COVID-19 vaccines affect human DNA.		
Not at all	464	59.9
Rarely	181	23.4
Occasionally	88	11.4
Very frequently	42	5.4
COVID-19 vaccines contain pig fat.		
Not at all	472	60.9
Rarely	181	23.4
Occasionally	78	10.1
Very frequently	44	5.7
A nurse fainted after she received the COVID-19 vaccine.		
Not at all	326	42.1
Rarely	305	39.4
Occasionally	106	13.7
Very frequently	38	4.9
COVID-19 vaccines contain live viruses that can make people sick with COVID-19.		
Not at all	413	53.3
Rarely	213	27.5
Occasionally	107	13.8
Very frequently	42	5.4
Those who have recovered from COVID-19 do not need to get vaccinated.		
Not at all	458	59.1
Rarely	171	22.1
Occasionally	97	12.5
Very frequently	49	6.3
Vaccines for COVID-19 have a microchip that can track the location of the patient.		
Not at all	465	60.0
Rarely	129	16.6
Occasionally	79	10.2
Very frequently	102	13.2
The COVID-19 vaccines are not safe because they were developed rapidly.		
Not at all	293	37.8
Rarely	198	25.5
Occasionally	135	17.4
Very frequently	149	19.2
The COVID-19 vaccine causes serious side effects like allergic reactions.		
Not at all	136	17.5
Rarely	296	38.2
Occasionally	185	23.9
Very frequently	158	20.4
The COVID-19 vaccine can cause infertility in women.		
Not at all	496	64.0
Rarely	202	26.1
Occasionally	55	7.1
Very frequently	22	2.8
Once you receive the COVID-19 vaccine, you will not have to wear a mask or practice social-distancing.		
Not at all	446	57.5
Rarely	153	19.7
Occasionally	84	10.8
Very frequently	92	11.9

**Table 5:** Risk perception about COVID-19.

	Total	
	*n*	%
The problem of the COVID-19 pandemic is important to me.		
Not at all	6	0.8
Slightly	4	0.5
Moderately	18	2.3
Quite a bit	65	8.4
Very much	237	30.6
Completely	445	57.4
I am worried that I may be infected with COVID-19 in the future.		
Not at all	11	1.4
Slightly	16	2.1
Moderately	37	4.8
Quite a bit	91	11.7
Very much	167	21.5
Completely	453	58.5
It is likely that I will be infected with COVID-19.		
Not at all	40	5.2
Slightly	98	12.6
Moderately	141	18.2
Quite a bit	196	25.3
Very much	140	18.1
Completely	160	20.6
I have felt at risk of COVID-19 infection.		
Not at all	120	15.5
Slightly	92	11.9
Moderately	106	13.7
Quite a bit	150	19.4
Very much	159	20.5
Completely	148	19.1

**Table 6:** Four domains of negative attitudes towards the vaccination programme. ^a^ Items were reverse coded.

	Total	
	*n*	%
Mistrust of vaccine benefits:		
I feel safe after being vaccinated. ^a^		
Strongly disagree	27	3.5
Disagree	26	3.4
Slightly disagree	85	11.0
Slightly agree	186	24.0
Agree	223	28.8
Strongly agree	228	29.4
I can rely on vaccines to stop serious infectious diseases. ^a^		
Strongly disagree	47	6.1
Disagree	53	6.8
Slightly disagree	113	14.6
Slightly agree	207	26.7
Agree	182	23.5
Strongly agree	173	22.3
I feel protected after getting vaccinated. ^a^		
Strongly disagree	22	2.8
Disagree	28	3.6
Slightly disagree	84	10.8
Slightly agree	200	25.8
Agree	229	29.5
Strongly agree	212	27.4
Worries about unforeseen future effects:		
Although most vaccines appear to be safe, there may be problems that we have not yet discovered.		
Strongly disagree	12	1.5
Disagree	25	3.2
Slightly disagree	105	13.5
Slightly agree	214	27.6
Agree	205	26.5
Strongly agree	214	27.6
Vaccines can cause unforeseen problems in children.		
Strongly disagree	64	8.3
Disagree	106	13.7
Slightly disagree	206	26.6
Slightly agree	195	25.2
Agree	122	15.7
Strongly agree	82	10.6
I worry about the unknown effects of vaccines in the future.		
Strongly disagree	59	7.6
Disagree	102	13.2
Slightly disagree	140	18.1
Slightly agree	216	27.9
Agree	130	16.8
Strongly agree	128	16.5
Concerns about commercial profiteering:		
Vaccines make a lot of money for pharmaceutical companies, but do not do much for regular people.		
Strongly disagree	160	20.6
Disagree	141	18.2
Slightly disagree	193	24.9
Slightly agree	143	18.5
Agree	67	8.6
Strongly agree	71	9.2
Authorities promote vaccination for financial gain, not for people’s health.		
Strongly disagree	335	43.2
Disagree	177	22.8
Slightly disagree	119	15.4
Slightly agree	92	11.9
Agree	28	3.6
Strongly agree	24	3.1
Vaccination programs are a big deception.		
Strongly disagree	457	59.0
Disagree	131	16.9
Slightly disagree	106	13.7
Slightly agree	63	8.1
Agree	10	1.3
Strongly agree	8	1.0
Preference for natural immunity:		
Natural immunity lasts longer than vaccination.		
Strongly disagree	173	22.3
Disagree	149	19.2
Slightly disagree	199	25.7
Slightly agree	128	16.5
Agree	69	8.9
Strongly agree	57	7.4
Natural exposure to viruses and germs gives the safest protection.		
Strongly disagree	272	35.1
Disagree	165	21.3
Slightly disagree	183	23.6
Slightly agree	95	12.3
Agree	38	4.9
Strongly agree	22	2.8
Being exposed to diseases naturally is safer for the immune system than being exposed through vaccination.		
Strongly disagree	279	36.0
Disagree	173	22.3
Slightly disagree	191	24.6
Slightly agree	93	12.0
Agree	24	3.1
Strongly agree	15	1.9

**Table 7:** Public confidence in the government. ^a^ Number for each item may not add up to a total number of study population due to missing values.

	Total	
	*n*	%
I am confident in the Malaysian government’s ability to effectively manage the COVID-19 vaccination program. ^a^		
1 (No confidence)	77	10.1
2	101	13.2
3	162	21.1
4	168	21.9
5	146	19.1
6 (Very high confidence)	112	14.6
I am confident in the ability of the Malaysian public health service to effectively manage the COVID-19 vaccination program. ^a^		
1 (No confidence)	32	4.2
2	61	8.0
3	119	15.5
4	181	23.6
5	203	26.5
6 (Very high confidence)	170	22.2

**Table 8:** Willingness to get vaccinated. ^a^ Number for each item may not add up to the total number of study respondents due to missing values.

	Total	
	*n*	%
If a COVID-19 vaccine is recommended for you, would you take it? ^a^		
No	8	1.0
Yes	756	99.0

**Table 9:** Results of regression models predicting four domains of negative attitudes towards the COVID-19 vaccine.

Variables	Mistrust of Vaccine Benefits				Worries about Unforeseen Future Effects				Concerns about Commercial Profiteering				Preference for Natural Immunity			
	Block 1		Block 2		Block 1		Block 2		Block 1		Block 2		Block 1		Block 2	
		*t*		*t*		*t*		*t*		*t*		*t*		*t*		*t*
Male (vs. female)	0.002	0.059	−0.02	−0.44	0.06	1.68	0.05	1.22	0.16	4.52 ***	0.14	4.09 ***	0.09	2.44 *	0.08	2.17 *
Age	0.23	5.38 ***	0.24	5.87 ***	0.17	3.84 ***	0.19	4.45 ***	0.21	5.16 ***	0.23	5.92 ***	0.22	5.16 ***	0.23	5.39 ***
Ethnicity (vs. other)																
Malay	0.37	1.94	0.37	2.02 *	0.57	2.98 **	0.56	2.95 **	0.31	1.70	0.31	1.77	0.06	0.32	0.05	0.26
Chinese	0.53	3.13 **	0.47	2.87 **	0.51	2.95 **	0.49	2.90 **	0.53	3.23 ***	0.47	3.00 **	0.18	1.05	0.16	0.93
Indian	0.18	2.40 *	0.17	2.42 *	0.20	2.75 **	0.22	3.06 **	0.15	2.14 *	0.15	2.29 *	0.04	0.56	0.05	0.63
Bumiputera Sabah/Sarawak	0.21	1.84	0.24	2.17 *	0.32	2.78 **	0.33	2.93 **	0.12	1.06	0.14	1.38	0.05	0.41	0.06	0.49
Income	−0.04	−0.95	−0.06	−1.41	−0.03	−0.79	−0.04	−1.07	−0.09	−2.29 *	−0.11	−2.91 **	−0.09	−2.04 *	−0.09	−2.22 *
Rural (vs. urban)	0.06	1.64	0.08	2.18 *	0.01	0.12	0.02	0.48	−0.01	−0.21	0.02	0.43	0.04	1.01	0.05	1.18
Employment (vs. private)																
Government	−0.06	−1.55	−0.05	−1.35	−0.01	−0.19	−0.01	−0.21	0.001	0.03	0.01	0.33	0.02	0.49	0.02	0.48
Self-employed (registered)	0.01	0.39	−0.004	−0.11	0.03	0.87	0.02	0.55	0.01	0.16	−0.01	−0.39	−0.02	−0.53	−0.03	−0.79
Self-employed (non-registered)	0.01	0.20	−0.001	−0.02	−0.05	−1.24	−0.05	−1.43	−0.03	−0.79	−0.04	−1.04	−0.03	−0.74	−0.03	−0.91
Unpaid	−0.03	−0.03	−0.03	−0.81	−0.04	−1.13	−0.05	−1.36	−0.02	−0.53	−0.02	−0.64	−0.01	−0.34	−0.02	−0.44
Not employed	0.06	1.57	0.04	1.19	0.01	0.18	0.01	0.29	0.01	0.17	−0.01	−0.18	0.00	−0.01	−0.003	−0.07
COVID-19 vaccine misinformation exposure	-	-	0.11	3.31 **	-	-	0.13	3.57 ***	-	-	0.10	2.96 **	-	-	0.12	3.21 ***
Perceived risk	-	-	−0.07	0.04 *	-	-	0.10	2.81 **	-	-	0.003	0.08	-	-	−0.003	−0.08
Confidence in government	-	-	−0.26	−7.07 ***	-	-	−0.12	−3.23 ***	-	-	−0.28	−7.98 ***	-	-	−0.09	−2.40 *
	Adj *R^2^* = 0.132 Δ*R^2^* = 0.078 *F*(16, 749) = 8.26 ***				Adj *R^2^* = 0.068 Δ*R^2^* = 0.044 *F*(16, 749) = 4.48 ***				Adj *R^2^* = 0.197 Δ*R^2^* = 0.082 *F*(16, 749) = 12.70 ***				Adj *R^2^* = 0.063 Δ*R^2^* = 0.022 *F*(16, 749) = 4.24 ***			

* *p* < 0.05. ** *p* < 0.01. *** *p* < 0.001.

## Data Availability

The data presented in this study are available on request from the corresponding author. The data are not publicly available due to ethical considerations.
